# Institutional Housekeeping

**Published:** 1911-12-02

**Authors:** 


### INSTITUTIONAL HOUSEKEEPING.

**(Contributions invited: Workers' Questions will be welcome.)**

### THE ART OF BUYING.

### 2. How to Get and Make Good Coffee.

Buyers inform us that in Great Britain the national taste for coffee is far below the average in other countries, and this is undoubtedly partly due to ignorance in the matter both of buying and preparing it.

The extremely poor stuff from which the cook is often expected to produce a fragrant brew would never be purchased at all did the housekeeper possess some elementary notions of choosing coffee.

The first point for purchasers to decide is whether or no they will have a pure coffee or a mixture of coffee and chicory. Opinions differ. Some experts oppose the mixture altogether. Others contend that when economy must be studied the admixture of chicory is advisable, since a finer beverage is obtained by adding a certain proportion of chicory to good coffee than by using low priced coffee by itself. It is legal for retailers to add chicory provided that the coffee is sold as a mixture and not as pure. In 1907 a discussion arose as to whether the use of chicory was unwholesome, and the *Lancet* summed up the controversy by stating that "Most dietetic authorities seem to agree that there is no reason for believing that chicory is in any way injurious to health." It may be noted here that chicory is the white fleshy root of the wild endive, which is washed, sliced, kiln-dried, roasted and ground. When chicory is bought separately and mixed at home in the desired proportion with the coffee, considerable care must be exercised in selecting it. To test a sample of ground chicory, a teaspoonful should be mixed with a cup of boiling water and allowed to draw. The liquor when tasted should be found to have a sweetish, slightly pungent, and not in the least disagreeable flavour. The usual proportion of chicory to coffee is about three ounces to a pound of coffee, or one spoonful to every four.

In choosing coffee the best test is the flavour, therefore the housekeeper must be at the pains to brew a cup from each sample after the most approved fashion. When ordering samples, it is most important to state whether the water in the district be hard or soft.

Roasting coffee is a delicate operation needing much experience and care to avoid scorching or over-roasting the berries, and to prevent smoke or fumes from reaching them. It is wise therefore to buy the berries ready roasted, and grind daily as required. When the ground coffee is mixed with chicory, great care must be exercised to blend the two substances thoroughly.

In spite of the law, ground coffee sold as pure may be discovered to be adulterated with chicory or with other roots, such as beetroot, parsnips or turnips. To test for chicory put a few grains of the sample on the surface of a wineglass of water, and almost immediately each particle will be surrounded by a yellowish-brown halo, which will be speedily diffused until the water is tinted. Pure coffee, if tested in a similar way, would make hardly any perceptible discoloration until about fifteen minutes had elapsed. The presence of other adulterants can be ascertained with the aid of a microscope, or by determining the specific gravity of the infusion.

When once coffee is roasted, it must not be exposed to the air, or it will lose many of its most valuable qualities. It should be stored in dry canisters or bottles, rendered as air-tight as possible, and as, like tea, it readily absorbs odours, it must not be kept in the proximity of strong-smelling articles. A moderately cool store cupboard not in the least damp, yet not intensely dry, is the best. Properly stored, unground coffee will improve by keeping for a fairly long period.

The main causes of failure in the preparation of coffee are: (*a*) the use of inferior coffee; (*b*) the use of stale coffee; (*c*) the use of water below boiling point; (*d*) the use of imperfectly cleansed pots; (*e*) the use of lukewarm milk. All these things must be guarded against, not once but as part of a daily system. The coffee mill must be carefully cleaned every day after use, as even a very little stale coffee left in from the previous day will spoil the flavour of the next brew. In damp weather the berries must be dried for a few minutes in the oven before being ground.

For café au lait, the usual amount allowed is one rounded tablespoonful to each half pint of water, or for large quantities, 2 oz. to every quart of water.

Heat an enamel or fireproof ware jug, put into it the coffee, add a small pinch of salt, and pour over the grounds the necessary amount of freshly boiled water. Stir the infusion well, cover the jug tightly and stand it in a pan of boiling water, over a gentle heat. After two minutes stir it again and leave it for about 8 to 10 minutes for the grounds to settle. Pour the coffee off through a very fine wire strainer, or one lined with muslin. After straining keep the coffee hot by standing the jug in a pan of boiling water. If a chicory mixture is used, it is best to heat the coffee just to boiling point, as this softens the chicory flavour.

A simpler method for large quantities is to tie the grounds in a very loose bag of muslin, and put them in an urn or perfectly clean saucepan, reserved for this purpose, in which is the requisite amount of boiling water. Leave long strings to the bag, which can be secured under the lid. Bring the water again to boiling point, then draw the pan aside and leave it by the side of the fire for ten minutes, when the coffee bag must be removed. Keep the coffee hot without reboiling it.

To serve the coffee always heat cups, coffee-pot, and milk-jug.

